# Natural language processing and machine learning to enable automatic extraction and classification of patients’ smoking status from electronic medical records

**DOI:** 10.1080/03009734.2020.1792010

**Published:** 2020-07-22

**Authors:** Andrea Caccamisi, Leif Jørgensen, Hercules Dalianis, Mats Rosenlund

**Affiliations:** a Department of Learning, Informatics, Management and Ethics, Karolinska Institutet, Stockholm, Sweden; b Department of Computer and Systems Sciences (DSV), Stockholm University, Stockholm, Sweden; c IQVIA Solutions Sweden AB, Solna, Sweden

**Keywords:** Clinical informatics, electronic medical records, machine learning, natural language processing, smoking, text mining

## Abstract

**Background:**

The electronic medical record (EMR) offers unique possibilities for clinical research, but some important patient attributes are not readily available due to its unstructured properties. We applied text mining using machine learning to enable automatic classification of unstructured information on smoking status from Swedish EMR data.

**Methods:**

Data on patients’ smoking status from EMRs were used to develop 32 different predictive models that were trained using Weka, changing sentence frequency, classifier type, tokenization, and attribute selection in a database of 85,000 classified sentences. The models were evaluated using F-score and accuracy based on out-of-sample test data including 8500 sentences. The error weight matrix was used to select the best model, assigning a weight to each type of misclassification and applying it to the model confusion matrices. The best performing model was then compared to a rule-based method.

**Results:**

The best performing model was based on the Support Vector Machine (SVM) Sequential Minimal Optimization (SMO) classifier using a combination of unigrams and bigrams as tokens. Sentence frequency and attributes selection did not improve model performance. SMO achieved 98.14% accuracy and 0.981 F-score versus 79.32% and 0.756 for the rule-based model.

**Conclusion:**

A model using machine-learning algorithms to automatically classify patients’ smoking status was successfully developed. Such algorithms may enable automatic assessment of smoking status and other unstructured data directly from EMRs without manual classification of complete case notes.

## Introduction

The use of electronic medical records (EMR) has been increasingly adopted in past decades and is used today in most industrialized countries for documentation of patient care (1). EMR data have become an important and integrated part of healthcare to facilitate the sharing of information between healthcare practitioners and document the care of patients, but can also be a source for epidemiological research and real-world evidence (RWE). However, data on many important patient attributes are typically only captured in free text fields as case notes in special sections in the EMR systems. Such unstructured properties of EMR data present an obstacle for RWE and clinical research.

The introduction of advanced analytics such as machine learning with text mining methods and algorithms offers the potential for more efficient use of unstructured EMR data for medical research (2–4). Developing algorithms for automatic classifications of patient characteristics, exposures such as smoking, or disease status in defined categories may allow for easier access to real-world data for studies of safety, effectiveness, and treatment patterns of pharmaceutical product use in routine clinical practice (5).

Most of the research specifically addressing the problems of classifying smoking status based on secondary data sources was conducted in conjunction with the 2006 ‘Smoking challenge’ announced by ‘Informatics for Integrating Biology and the Bedside’ (i2b2), a Centre for Biomedical Computing funded by the National Institute of Health in USA, and by those who continued building on that work (5–7). In addition, a recent US study on dental health records developed a similar model as presented here, but focussing on tobacco consumption (8). However, despite the recent advancements in text mining and machine learning, making use of unstructured information from EMRs such as smoking status still presents a challenge for researchers. The aim of this project was to develop a text mining model using machine-learning techniques to classify the smoking status of patients using EMR data and to assess the performance attributes of the best machine-learning model compared to a rule-based model. Here, we describe the process to derive the best machine-learning model and how it compares with a manual classification.

## Methods

The software tools used for pre-processing data were Microsoft Excel and Notepad++, while the Waikato Environment for Knowledge Analysis software (Weka) was used for text mining tasks including text classification and analysis (9).

### Smoking classes

We used EMR data including patient level smoking information from two observational studies collected during 2014 and 2013 (10,11). The following smoking definitions were applied, which were also the most common classes used in the ‘Smoking challenge’ (5):

- *Current smoker:* Records of explicit statements or details leading to the *obvious* conclusion that the patient is smoking cigarettes, cigars, or pipe (e.g. statements like ‘10 cigarettes/day’; ‘Yes, smokes’). Any explicit smoking consumption was enough to be classified as current smoker regardless of quantity or frequency. Short answers like ‘Yes’ were coded as unknown if the text field was derived from the tobacco–alcohol fields of the EMR, since it was not possible to distinguish if it referred to smoking or alcohol habits.
- *Ex-smoker:* Records which explicitly state that the patient used to smoke but is currently not smoking, regardless of the time since the patient had stopped smoking (e.g. ‘Stopped smoking’; ‘Smoke free since 1998’; ‘Ex-smoker’).
- *Non-smoker:* Records which explicitly state that the patient is not a smoker (e.g. ‘Doesn’t smoke’; ‘Non-smoker’; ‘Never smoked’). Short answers like ‘No’ were coded unknown if the text field was derived from the tobacco–alcohol fields of the EMR, if impossible to distinguish smoking or alcohol habits.
- *Unknown:* Records which do not fit in any of the previous classes (e.g. ‘Didn’t ask’; ‘136/72 Pulse 70’).

As the purpose of this project was to capture data on smoking habits only, any patient records of snuff without any evidence of smoking was coded as unknown (snuff is a moist tobacco powder commonly used in Sweden) (12,13).

### Datasets

**Figure 1.**
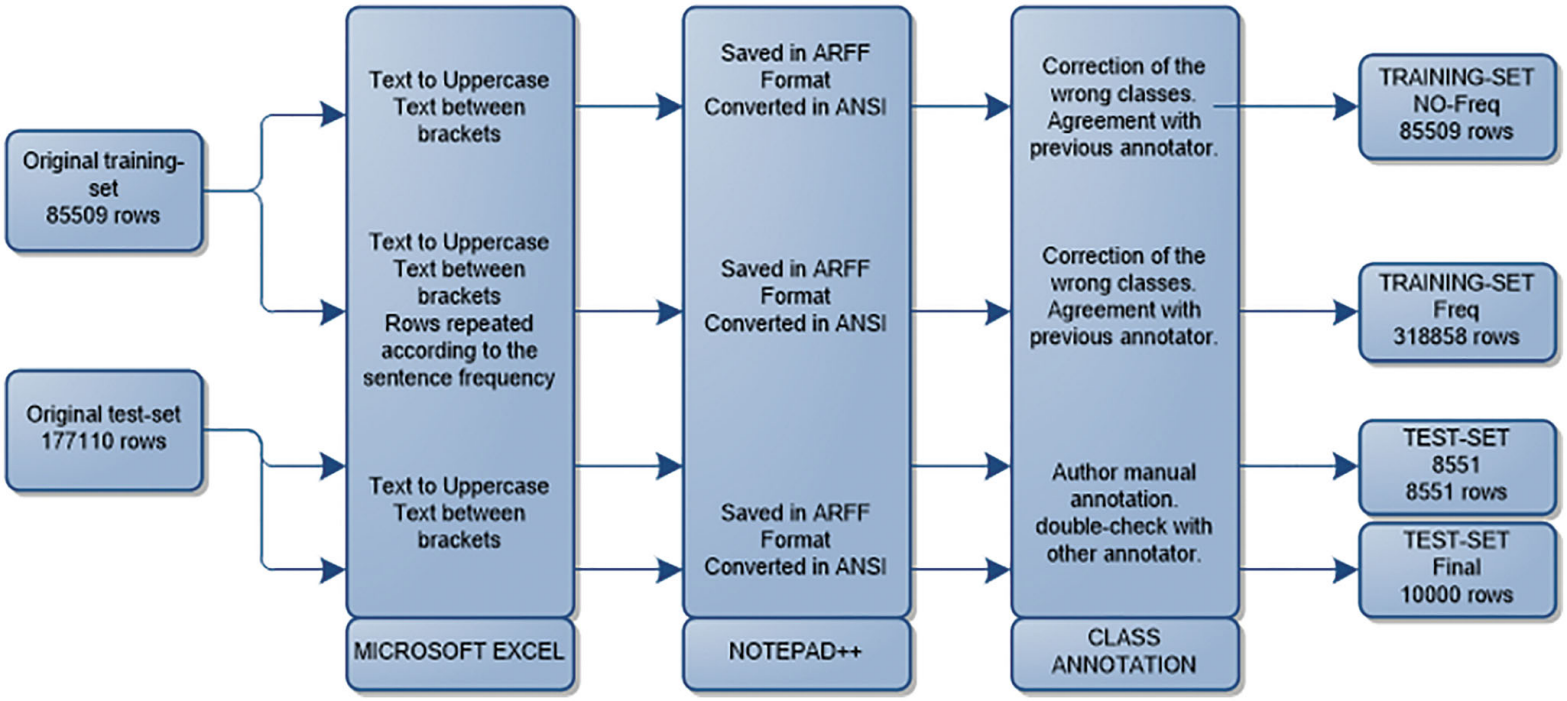
Description of the workflow used to pre-process data, to obtain coherent and manually classified training-sets and test-set.

First, the data were anonymized and cleaned from all patient information other than the smoking status text field. We then created different datasets to train and test the models (Figure 1). The first training dataset (‘NO-Freq’) was derived from 2014 EMR data and consisted of 85,509 rows and 3 columns where smoking information was present, with the first column indicating that the row belonged to a smoking status text field, the second containing the actual text typed in the field, and the third described how many times the same sentence appeared in the dataset (sentence frequency). A second training dataset (‘Freq’) was generated from the same study data and included 318,858 rows by repeating the rows according to the sentence frequency. This dataset was used to evaluate if sentence frequency may affect the classification when training the models, i.e. how well the classification correctly discriminates between the smoking classes. That may occur in cases where two similar sentences belong to different classes, because using sentence frequency dramatically increases the term-frequency of the words in the most common sentences of the dataset. That in turn influences measures such as the information gain and thus impact the classifiers, particularly for decision trees (14,15). This dataset was used to train all models. The proportion of smokers in our datasets was coherent with national statistics of smoking status in Sweden (16).

We then created two test datasets based on the 2013 study data. The first test dataset included 8551 rows out of a total of 177,000 rows from that study data and was used to test all derived models. The second test dataset included another subset of 10,000 rows from the same study data and was used to compare the performance of the best performing model and the rule-based model. We decided to use a different test-set (i.e. not a 10-fold cross-validation commonly applied in data mining) because of the availability of a large amount of data allowing us to test the models on unseen data. This test data contained up to 603 characters and an average of 24.5 characters per sentence for the fields with smoking status information, while the original training dataset included up to 84 characters per sentence (average 45), with 90% of the sentences appearing only once.

The training dataset was double-coded manually, i.e. an additional annotator went through the file correcting any errors and agreeing with the previous annotator on all proposed changes if in agreement. Both test datasets of 8551 entries and 10,000 entries were assessed by another annotator to agree on a gold standard. Any mismatches were jointly discussed, and a common agreement was made to achieve a 100% match of those sentences.

### Model development

The model development was based on the variation of different macro-settings of four classification algorithms with the tool ‘Weka’. We considered the following four classification algorithms: Sequential Minimal Optimization (SMO), k-NN, Naïve Bayes, and J48. SMO is an iterative algorithm used to solve the quadratic programming problem during support vector machine (SVM) training, which divides the problem into smaller sub-problems in order to find the hyperplane with the maximum margin (17). The k-NN is a classification algorithm which assigns the class for majority vote depending on the class of the *k* nearest neighbours to a test sample (18). Naïve Bayes is a probabilistic classification algorithm based on Bayes’ theorem which assumes a high independence between the training-set attributes (19). J48 is the Weka implementation of the more known C4.5 classification algorithm. The algorithm creates a binary decision tree checking iteratively for each tree node the information gain ratio of every attribute, in order to evaluate which one to split on (20). SMO was chosen because it performed best in the ‘Smoking challenge’, and we included k-NN and J48 because they both performed well in that challenge (5,21,22), while Naïve Bayes was considered because it is an algorithm which is robust to irrelevant features and works well especially in situations with unbalanced classes (23,24). The classifier-specific settings in Weka were kept as default, as only the macro-settings (sentence frequency, classifier type, tokenization, and attribute selection) were of interest. All Weka settings have been summarized in Table 1.

**Table 1.** Summary of features and weka settings used to create the models.

Feature	Option/setting
Sentence frequency	Yes No
Classifier	SMO k-NN Naïve Bayes J48
Attribute selection	Yes No
Tokens	Unigrams Unigrams + bigrams
Classifiers settings	All classifier specific settings were kept as default except for k-NN *k* value which was set to 1 To determine the optimal *k*, a 10-fold cross-validation was run on both training datasets ‘Freq’ and ‘NO-Freq’, both for unigrams and unigrams + bigrams, testing *k* equal to 1, 3, 5, and 10
SelectAttributes settings	Attribute Evaluator was set to ‘InfoGainAttributeEval’ Search Method was set to ‘Ranker’ The Ranker setting threshold was set to 0.0 in order to discard attributes with a negative Information Gain All other settings were kept as default
StringToWordVector settings	‘WordsToKeep’ was set to 15,000 in order to take into account all the single word tokens as attributes The tokenizer was set on ‘Word tokenizer’ for unigrams or ‘N-gram tokenizer’ with minimum size equal to 1 and maximum size equal to 2 for unigrams + bigrams, depending on the chosen model All other settings were kept as default

In text mining, tokenization is the act of breaking up a sequence of strings into pieces such as words, and we applied two different models for tokenization in order to structure the data by transforming the original text string into a collection of word vectors and binary values (25), one using unigrams (single words) and another combining unigrams and bigrams (single words or two consecutive words). In Weka, when the ARFF file (Weka format of input) is loaded, only ‘text’ and ‘SmokingStatus’ are present as attributes, and the filter ‘StringToWordVector’ was used, as it scans the rows in the training-set and creates tokens which become the attributes, according to the specified settings. Thus, a word vector was created for each training-set row which contained the binary value 1 corresponding to an attribute present in that sentence or 0 otherwise.

When the training-set was tokenized and the attributes ready, we created another test selecting a lower number of attributes in the training-set to reduce dimensionality. To select only the attributes with a higher predictive power, the Weka filter ‘SelectAttributes’ was used. The algorithm used for the attribute selection was the ‘InfoGainAttributeEval’ with the ‘Ranker’ function since it demonstrated good performance overall using different classifiers on diverse datasets (26). The Ranker setting threshold was set to 0.0 to discard attributes with a negative information gain.

After the previous tests were executed in Weka, 32 models were produced. To evaluate and compare the models, several statistical measures were used, including positive predictive value (PPV) (true positives/[true positives + false positives]), sensitivity (true positives/[true positives + false negatives]), F-score (2 × [PPV × sensitivity]/[PPV + sensitivity]), accuracy ([true positives + true negatives]/[true positives +true negatives + false positives + false negatives]), and receiver operating characteristic (ROC) area (27). The ROC curve is a plot of sensitivity on the *y* axis and false positive rate (FPR) on the *x* axis (27). It is an effective method for the evaluation of the performance, where a higher ROC area indicates a better performing model. An area under the ROC curve equal to 1 is considered the perfect case since it means the FPR is 0 and the sensitivity is 1 (27). Finally, in the second part of the study, the Error cost was used as discriminating factor, which is defined as the cost assigned to each kind of sentence misclassification based on the expected relative importance.

F-score and ROC area were calculated by Weka and consisted of the weighted average of the individual class measures, while accuracy was calculated dividing the correctly predicted number of records by the total number, independently by the class-specific accuracy.

**Figure 2.**
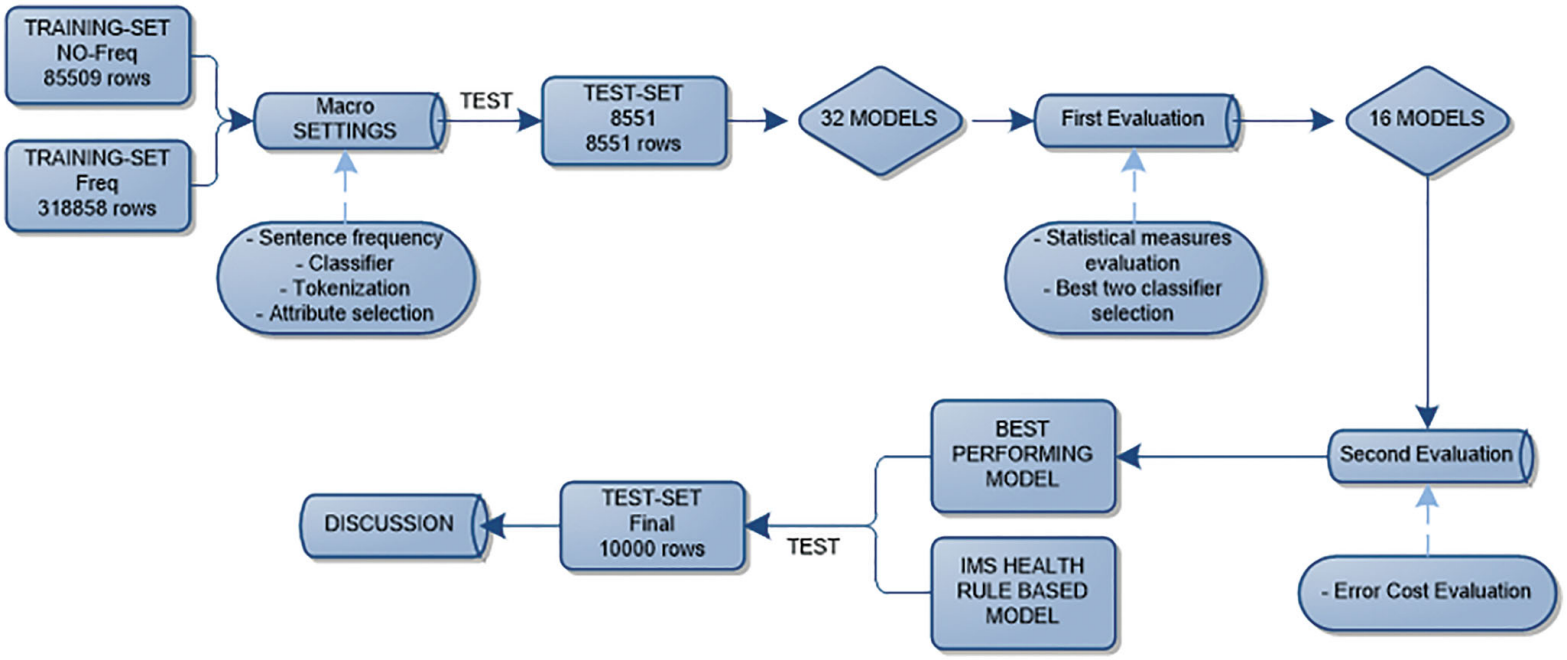
Evaluation workflow.

All 32 models were evaluated on the test dataset (4 methods with 8 options) with 8551 entries (Figure 2). The test datasets were created semi-randomly, i.e. about 20,000 entries from the original test-set were manually classified and randomly picked by classes and narrowed down to 8551 and 10,000, recreating the same class distribution as in the sentence frequency dataset. This was done to obtain more realistic results that would make the model more relevant for future use (Table 2).

**Table 2.** Training-set class distribution.

	Training-set NO-Freq		Training-set Freq	
	Number	Percent	Number	Percent
Current smoker	40,743	47.6%	77,401	24.3%
Non-smoker	8217	9.6%	185,456	58.1%
Ex-smoker	28,694	33.6%	38,791	12.2%
Unknown	7855	9.2%	17,210	5.4%
Total	85,509	100%	318,858	100%

The models were built with the two best performing classifiers (*n* = 16). They were then compared using an error cost matrix, in which a weight was given to the different combinations of possible types of misclassification to account for the between-class hierarchy (e.g. an ex-smoker classified as smoker would be considered a less serious error compared to a smoker classified as a non-smoker). We considered the hierarchy of decreasing importance for the error in the following sequence: YES→NO and NO→YES, followed by NO→EX and EX→NO, and then YES→EX and EX→YES. A direction of misclassification from any of the smoking classes to unknown was assumed to only decrease the statistical power, while misclassifying an unknown record to any of the smoking classes might introduce bias and hence were assigned a double cost. The cost of each type of misclassification was then multiplied by the values in the defined cost matrix as described in Table 3, to obtain the model total cost. The model with the lowest cost or error was considered the best performing model, regardless of its accuracy. The best model and the original rule-based model were compared by testing their individual performance on a final test dataset as described in the workflow in Figure 2. The rule-based model was constructed based on expert opinion using a manual classification of all combinations of smoking in the text fields.

**Table 3.** Error cost matrix with assigned weights to each type of prediction misclassification.

	Manually classified as			
Predicted as	Smoker	Non-smoker	Ex-smoker	Unknown
Smoker	0	20	5	1
Non-smoker	20	0	10	1
Ex-smoker	5	10	0	1
Unknown	2	2	2	0

### Ethical considerations

Data for this study received ethics approval from the authorized Ethical Review Board (ERB) (Dnr.2014/54–31/3 and Dnr.2013/267–31/3) (10,11).

## Results

The inter-annotator agreement was 99.9% (958 records classified differently out of 104,060) on both the training and test datasets after the individual classification. There were 85,509 text strings containing any information of smoking in the EMRs, which were classified as smoking in 40,743 entries, as non-smoking in 8217 entries, and as ex-smoking in 28,694 entries (Table 2). There were in total 318,858 entries of smoking-related sentences in the dedicated EMR fields, which were classified as 77,401 smoking, 185,456 non-smoking, and 38,791 ex-smoking. The sentence ‘No’ occurred in 93,358 different records of smoking text fields and appeared in an equal number of rows in the second training-set, while only once in the first. The proportion of text strings that could not be manually classified (Unknown) was 9.2% for the first training-set and 5.4% for the second.

**Figure 3.**
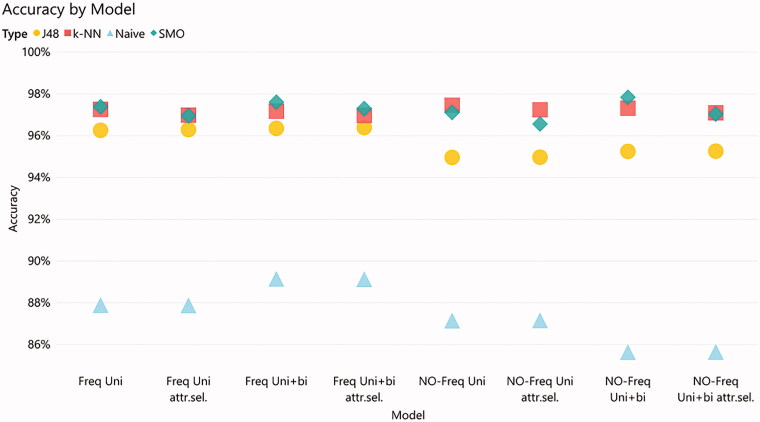
Accuracy of the models. The abbreviation ‘Uni + bi’ refers to the utilization of a combination of unigrams and bigrams as features in the training-set. The abbreviation ‘Attr. sel.’ refers to the selection of the most relevant attributes in the training-set.

Ranking all 32 models run on the test dataset showed that the SMO model with no frequency and both unigrams and bigrams as tokens achieved the highest accuracy, followed by the same classifier with the frequency feature combined with both unigrams and bigrams as tokens (Figure 3). The third best accuracy was achieved with the k-NN model with no unigrams and bigrams, followed by the same classifier with no frequency but unigrams only. For the J48 models, the highest accuracy was achieved with the features frequency, unigrams/bigrams, and attribute selection, followed by the one with frequency and unigrams only. The Naïve Bayes models which performed the worst, displayed the highest accuracy with frequency and unigrams/bigrams features, followed by the same features together with attribute selection.

Models with SMO and k-NN classifiers had an average accuracy of 97.22% and 97.19%, F-score of 0.972, and ROC area of 0.988 (Table 4). Models with the J48 classifier displayed a similar ROC area result as the SMO and k-NN models, but noticeably lower accuracy and F-score. The Naïve Bayes models presented the lowest results on average on all measures in comparison with the other classifiers.

**Table 4.** Average of the statistical measures of the models by classifier.

	Model			
Classifier	SMO	Naïve Bayes	k-NN	J48
F-score	0.972	0.879	0.972	0.959
ROC area	0.988	0.961	0.988	0.989
Accuracy	97.22%	87.45%	97.19%	95.72%

Applying the cost matrix described in Table 3 to the confusion matrix of the models with the two best classifiers based on the assessment above (i.e. SMO and k-NN) demonstrated that the lowest cost of the remaining 16 models was scored by the SMO classifier and both unigrams and bigrams as tokens, without any selection based on the information gain (Table 5).

**Table 5.** Result of the application of the cost matrix to the remaining 16 models.

	Classifier			Feature		
Model	SMO	k-NN	Freq	Unigrams only	Attribute selection	Cost
1	✓	–	–	✓	–	1751
2	✓	–	–	✓	✓	1983
3	✓	–	–	–	–	1059
4	✓	–	–	–	✓	1467
5	✓	–	✓	✓	–	2139
6	✓	–	✓	✓	✓	2322
7	✓	–	✓	–	–	1627
8	✓	–	✓	–	✓	1921
9	–	✓	–	✓	–	1949
10	–	✓	–	✓	✓	1990
11	–	✓	–	–	–	1970
12	–	✓	–	–	✓	1873
13	–	✓	✓	✓	–	2182
14	–	✓	✓	✓	✓	2327
15	–	✓	✓	–	–	2060
16	–	✓	✓	–	✓	2073

Comparing the performance of the best model and the rule-based model on the final test dataset demonstrated that the machine-learning model achieved a higher PPV (98.10%), sensitivity (98.10%), and F-score (0.98), with an accuracy of 98.14% compared to the rule-based model which had an accuracy of 79.32% (Table 6). To maximize the new model performance, the macro-settings which need to be adopted are the SVM SMO classifier and the unigrams + bigrams tokenization. Sentence frequency and attributes selection did not improve the model.

**Table 6.** Statistical measures of the best model and the rule-based model.

Measure	Best model	Rule-based
Positive predictive value (PPV)	98.10%	79.90%
Sensitivity	98.10%	79.30%
F-score	0.981	0.756
Accuracy	98.14%	79.32%

## Discussion

We developed an algorithm to enable automatic classification of smoking status based on patients’ EMR data using machine-learning techniques. Our results demonstrated better performance compared to a rule-based model, with close to 20% improved accuracy on the same test dataset. Thus, our study provides further understanding of how to make use of unstructured EMR data for large-scale real-world evidence and epidemiological research by applying more efficient modern machine-learning techniques and more specifically adds to the literature of how to automatically classify smoking status for patients using secondary data sources.

Most of the previous work addressing the methods for classifying patients’ smoking status has been conducted as a result of the ‘Smoking challenge’ (7,21,22,28–32), and by others who continued building on that work (5,6,33). A recent US study on dental health records developed a similar model based on machine learning (8). However, to our knowledge, there is no such prior work performed on Swedish data. In accordance with the ‘Smoking challenge’, we applied the most frequently used smoking categories, i.e. ‘Current smoker’, ‘Past smoker’, ‘Non-smoker’, ‘Unknown’, while some have also considered a more generic class of ‘Smoker’ (5). Similarly to previous publications on this topic, we took advantage of the most commonly used text mining tool ‘Weka’ (9), which is expected to make our results and model development described here more relevant also for others.

An SVM was also used as classifier in previous smoking classification studies, including the two Health Information Text Extraction (HITEx) studies (2,5), and in the best model from the ‘Smoking challenge’ (5), by Clark et al. (30). They achieved 90% accuracy and 0.83 F-score, and 96% accuracy and 0.90 F-score, respectively, while the model in our study achieved an accuracy of 98.14% and an F-score of 0.981. The results obtained by those two models can be directly compared to the ones obtained in the current study since the models entail the same action of classifying a sentence regarding the patients’ smoking status. However, some differences should be acknowledged. For instance, in the two previous models, the text was collected from different parts of the EMR and different techniques were used to isolate the sentences relevant for the patients’ smoking status. In this study, however, only the information in the ‘smoking’ or ‘smoking/alcohol’ field of the EMR case notes was used. In addition, the text language in the two previous models was English, which may influence the effectiveness of a setting over another—for instance, tokenization with unigrams, bigrams, or trigrams could have a more or less enhanced difference in results depending on the structure of the adopted language. Also, the training-set of the two previous models was tokenized using unigrams only, and in the ‘Smoking challenge’ the EMRs where annotated by two pulmonologists. In the ‘Smoking challenge’, 398 annotated EMRs of complete case notes were used in the training-set, whereas 85,509 (only smoking text field) were used in our study.

The performance of the classifiers used in this work can be compared with the results obtained in previous studies and with the expectations related to their classifier-specific characteristics. SMO appeared more valid and reliable as classifier for the automated smoking status classification in this study as well as in the ‘Smoking challenge’, with a higher accuracy and F-score because of its characteristics of being able to perform better with a large quantity of input data (34). In fact, SVM SMO divides the initial quadratic programming problem into the smallest possible sub-problems and solves them analytically one by one. It allows the algorithm to always converge without regard of the dimensionality (17). A recent study on dental health records from the US found that SVM performed best to classify patients according to smokers, non-smokers, and unknowns, with a PPV and sensitivity of 98% and F-score of 0.98 (8). In addition, that study included an assessment of the patient’s tobacco consumption, but it was limited to three classifiers instead of four as in our study and there was no consideration of the cost matrix.

The k-NN classifier performed better than expected in our study. The *k* value was set to 1, thus the used classifier was the nearest neighbour (NN). Good results achieved using the NN classifier imply the almost total absence of outliers in the training-set (18). This could be due to the orderly structure of the Swedish EMRs which contain a specific text field for the smoking status (1). On the other hand, Naïve Bayes was the simplest of the four algorithms used, and it can achieve better results on fewer data (20), especially with unbalanced classes (23), since it is robust to irrelevant features (24). Our results, however, illustrate that with a larger amount of data, Naïve Bayes does not achieve as high accuracy as the other algorithms. J48 performed better than in the ‘Smoking challenge’, probably also because of the orderly structure of the Swedish EMRs with a specific smoking text field (1). On the other hand, when training the algorithm, the quickest classifier was NN (min. 5 s) followed by Naïve Bayes, SVM SMO, and J48 (up to 7 days), while the quickest classifier during testing was J48 (1 s minimum) followed by SVM SMO, NN, and Naïve Bayes (up to 20 min).

All the 32 models we developed using machine learning, even the worst-performing one using the Naïve Bayes classifier which achieved an accuracy of 85.63%, appeared better than the rule-based SAS model which reached a 79.32% accuracy. This confirms that if enough training data are available, a machine-learning classification model generally has the capability of performing better than the analogue rule-based model (35,36). This is mainly due to the increasing difficulty of creating a comprehensive rule-based model as the size and diversity of the dataset increase. However, one of the issues with developing algorithms to capture data from case notes is the risk of grammatical errors and typos in the text and the presence of diverting sentences, e.g. ‘The mother is a heavy smoker’ or ‘The father smoked for 20 years’, which complicates the development of a rule-based approach to define which sentence is related to the current patient.

After a first analysis of the 188 sentences misclassified by the best model in this study out of the 10,000 in the ‘Final test-set’, the most common mistakes were related to other tobacco types such as snuff. For example, the model appears to give a considerably higher priority to ex-smoker class keywords compared to the unknown class keywords, which is likely caused by the fact that sentences regarding non-cigarette tobacco such as snuff are not always considered unknown since a reference to smoking can be present as well, while keywords used for ex-smokers are mainly adopted for that class only. This means that a sentence referring to a patient who had stopped using snuff in December (original text string: ‘Slutade snusa i december’) may be wrongly classified as ex-smoker because of the presence of ‘stopped’, regardless of the presence of any reference to snuff use. That may be caused by low numbers of examples of ex-snuff consumers in the training-set. Another situation involves sentences with combinations of tobacco–alcohol text fields with discordant evidence about tobacco and alcohol or reference to alcohol only. Correspondingly, if the model gives a higher priority to a keyword referred to the alcohol consumption, compared to the keyword referred to the smoking habit, a sentence such as ‘Doesn’t smoke, wine sometimes’ (original text string: ‘Röker inte, vin ibland’) may be misclassified as ‘smoker’ because of the word sometimes. For the same reason, in cases where there is only a reference to alcohol present in the text field, a sentence such ‘1 glass of wine sometimes in the weekend’ (original text string: ‘1 glas vin ibland på helgen’) may be misclassified as ‘Smoker’. Additionally, the single word sentence ‘Not’ was classified as Unknown by the model even though it was classified as ‘No’ in the training-set. This misclassification could be due to different reasons such as the scarce presence of the two words in the training-set, their combination with other words in longer sentences, or because the model considers their absence more relevant than their presence. Nevertheless, assessing all possible causes of this misclassification was not within the scope of this work. Another example involves sentences with the character ‘/’ between two words. Sentences such as ‘Party smoker/Moderate’, in this case referring to smoking/alcohol habits, may be misclassified as ‘Unknown’ as the tokenization process did not remove the ‘/’ character and therefore it was considered as a single word. This misclassification could be avoided by removing the ‘/’ character; however, sentences such as ‘10 cigarettes/day’ are correctly classified independently of the number present in the sentence, precisely because of the presence of this character. The last example involves sentences which would have been interpreted with difficulty even by a human reader. For instance, the exact meaning of the sentence ‘Smoked 10–20 cig for 45 years. Stopped for 3 years but started again after 2 years and smoked for about 1 year, now stopped for some weeks’ may be difficult to understand, and such sentences may need to be read more than once. With this kind of sentence, the model would make a prediction which in most of the cases is expected to be wrong, due to the presence of repeated contrasting keywords.

It is important to consider the impact of such measurement error and misclassification of covariates based on machine learning when used in regression models in epidemiological studies of exposure–disease associations. Methods to account for bias due to misclassification of exposure covariates such as smoking have been described by others (37). A sensitivity of 79.3% in a rule-based classification means that in a study of 10,000 patients we would misclassify 621 smokers as non-smokers, including 518 smoking cases and 104 controls misclassified as non-smokers. The improvement by applying our algorithm with a sensitivity of 98.1% means that we would only misclassify 57 smokers as non-smokers, including 48 smoking cases and 10 controls misclassified as non-smokers. Assuming a smoking prevalence of 30% and a true odds ratio (OR) for smoking-related lung cancer of 9.00 would translate into an OR of 8.85 and 7.63 using these models, respectively, which is of similar magnitude as other simulations (38). This was assuming a non-differential misclassification, but if the likelihood of being classified as a smoker depends on disease status, e.g. if lung cancer patients were more likely to report historical smoking, the misclassification would cause overestimation of the relative risk. Such fixed-parameter-bias sensitivity analyses are, however, simplistic, and probabilistic bias analysis or Bayesian analyses are recommended for risk assessment to account for all sources of uncertainty (39).

Limitations of this study include that the algorithms have been applied on a rather short text field where smoking information is entered. Moreover, the models were selected only using macro-settings (sentence frequency, classifier type, tokenization, and attribute selection). However, each classifier has its specific parameters which for the purpose of this study were not considered and therefore kept as default values in Weka (except for k-NN *k* value). A selection of the optimal values for these settings (parameters optimization), using for instance a grid-search algorithm, could have further improved the models’ accuracy (37). In addition, multiple local language speaking annotators might have further decreased any possibility of human misclassification in the test-set or in the training-set. Thus, further improvements might be possible by finding the optimal classifier-specific parameters for the best performing model. The SVM SMO classifier used in the best model has as specific settings: the exponent of the polynomial kernel and the complexity value ‘C’ which is set to 1 by default. ‘C’ is a trade-off value between the classifier generalization and the training error. Hence, further improvements might be possible through optimization of ‘C’, since the complexity parameter is a positive number between zero and infinite (40). Another possibility would be to use innovative techniques like ‘deep learning’, which consists of a convolution of artificial neural networks with a high number of hidden layers (41). That appears promising for data mining, but the scientific body of evidence may still be limited.

In conclusion, the machine-learning model performed best when using the SVM SMO classifier and selecting both unigrams and bigrams in the training-set, with an accuracy of 98.14% compared to 79.32% using a rule-based model on the same test-set. These results illustrate the possibilities of using machine-learning techniques for automatic health-related text classification in EMRs, enabling the transformation of unstructured information to structured format with good accuracy.
